# From IBS to DBS: The Dysbiotic Bowel Syndrome

**DOI:** 10.1177/2324709616648458

**Published:** 2016-05-09

**Authors:** Peter Benno, Atti-La Dahlgren, Ragnar Befrits, Elisabeth Norin, Per M. Hellström, Tore Midtvedt

**Affiliations:** 1 Läkarhuset Hötorgscity, Stockholm, Sweden; 2 Karolinska Institutet, Stockholm, Sweden; 3 Uppsala University, Uppsala, Sweden

**Keywords:** irritable bowel syndrome, microbiota, postinfectious IBS, diarrhea, pain, dysbiosis

## Abstract

Irritable bowel syndrome is a chronic gastrointestinal disorder characterized by abdominal pain and altered bowel habits in the absence of organic disease. We present 2 cases where diarrhea-predominant irritable bowel syndrome occurred in association with earlier intestinal infection or antibiotic treatment. Both were successfully treated with instillation of an anaerobic cultivated human intestinal microbiota. Thereafter, they were symptom free for at least 12 months. We now introduce the term *dysbiotic bowel syndrome* covering cases where a disturbed intestinal microbiota is assumed to be present. We recommend that restoration of the dysbiotic gut microbiota should be first-line treatment in these conditions.

## Introduction

Irritable bowel syndrome (IBS) is a clinical entity that affects up to 11% in different communities.^1^ The condition is leading to low quality of life and may render patients with considerable disabilities. The etiology and underlying pathophysiology is assumed to be multifactorial. This is reflected by numerous treatment options, most often with suboptimal clinical results.

There is now a growing body of evidence indicating that disturbances in the gut microbiota might play a role in the development of IBS.^2^ The basic concept for a disturbance of the gut microbiotic ecosystem, so-called dysbiosis, is that about 10% of patients who once had an intestinal infection develop postinfectious IBS.^3^ In such cases, locally acting antibiotics such as rifaximin may have a positive, but transient, therapeutic effect,^4^ whereas broad-spectrum antibiotics may increase the risk of developing IBS.^5^

Herein we present 2 IBS cases, both of which fulfilling the Rome III criteria,^6^ which on solid grounds were assumed to be commenced and maintained due to a dysbiotic gut microbiota. Both patients were cured with normalized bowel habits after treatment with an anaerobically cultivated human intestinal microbiota.

## Methods

The microbiota transplant that was used originates from fresh fecal matter collected in 1994 from a healthy donor of Scandinavian descent on ordinary Western diet.

The subject and feces were investigated for relevant pathogens.^7^ Over the years the fecal microbiota has been recultivated under strict anaerobic conditions at 1- to 2-week intervals.^8^

Using lidocaine spray (10 mg/mL; AstraZeneca, Södertälje, Sweden) as local anesthetic for the pharynx, the esophagus was intubated with a standard gastroscope (Olympus GIF-Q 180; Shinjuku-ku, Tokyo, Japan). The instrument was inserted for delivery of the microbiota in the descending part of the duodenum. After removing the instrument the patient was allowed to rest for 15 minutes before returning home.

## Case Report

### Case 1

The patient was a 32-year-old woman, previously appendectomized and intermittently using nonsteroidal anti-inflammatory drugs for a whiplash injury. Four months earlier, she had a sudden onset of abdominal pain and watery diarrheas without blood up to 20 times per day. On querying her epidemiological history, it was revealed that 4 months earlier she had been on a 10-day course of flucloxacillin (500 mg, 3 times daily) for a bacterial skin infection. This led to hospitalization for rehydration and investigation. A thorough workup, including endoscopic examinations and repeated microbiological stool analyses for *Salmonella, Shigella, Campylobacter*, and *Yersinia* spp were all negative. Analysis for *Clostridium difficile* toxin was also negative as were parasites and ova. Colon biopsies revealed no pathology. Besides a low serum albumin (31 g/L), her blood count and chemistry were normal.

Treatment with metronidazole (400 mg, 3 times daily) and cholestyramine had no effect and later rifaximin (550 mg, 3 times daily) had no effect, each treatment course given for 14 days in a row. Symptomatic treatment was added with codeine 25 mg 3 times daily and amitriptyline 50 mg at night. In spite of this, she still had 5 to 6 urgency stools per day and was also incontinent at times. Her symptom burden required halftime sick leave from work. She requested a “fecal transplant” and received 3 treatments with an anaerobic cultivated human intestinal microbiota during the following month. The cultured microbiota was infused through a gastroscope in the descending part of the duodenum. After this, she slowly recovered and within weeks all drug treatments were discontinued as her gut function improved to 1 or 2 solid stools per day.

Fourteen months later, she was diagnosed with endometritis. Treatment was given with phenoxymethylpenicillin (1 g, twice daily) for 10 days. Within a few days, she again developed diarrhea and abdominal pain, which required codeine for symptom relief. Tests for *C difficile* toxins were all negative. We then decided to give another 2 treatments with the cultured intestinal microbiota she had received before. Again, symptoms improved promptly and codeine treatment was discontinued. Follow-up 1 year later showed that our patient took no medicines and had normal stool frequency.

### Case 2

The patient was a 25-year-old male, previously healthy globetrotter with intermittent minor lower back pain. During a journey to Australia, he acquired a gastrointestinal infection with up to 10 loose stools per day and abdominal pain.

Stool investigations revealed *Blastocystis hominis, Entamoeba coli, Dientamoeba fragilis, Endolimax nana*, and *Giardia lamblia*. He was treated with multiple antibiotic courses including metronidazole (400 mg, 3 times daily) but was only temporarily relieved even with negative stool investigations. On returning to Sweden, diarrheas returned as did his abdominal pain. One year later he was referred to the clinic of infectious medicine and thorough examinations showed no etiological factor, resulting in a diagnosis of postinfectious diarrhea-predominant IBS (IBS-D). Separate treatments with cholestyramine and loperamide had no effect, nor had antibiotics that were prescribed based on suspicion of small intestinal bacterial overgrowth. The patient requested treatment with the cultured microbiota as in Case 1. Follow-up 4 weeks later showed normalized bowel habits with solid stools every 1 to 2 days. On follow-up 6 months later his bowel habits had returned to normal.

## Discussion

Both these patients fulfilled the criteria for IBS-D. Most likely, their chronic disease was caused by gastrointestinal dysbiosis; In Case 1, it was initiated in conjunction with antibiotic treatment, and in Case 2, it evoked the infestation itself or the following antibiotic treatment.

In order to evaluate possible mechanisms underlying some of the symptoms and findings in IBS-D, such as abdominal pain, diarrhea, altered intestinal motility, abdominal distension, and gas production, it is relevant to mention that all these conditions might be influenced by variations in some intestinal host-microbe or microbe-microbe interactions, such as production of and sensitivity to neurotransmitters,^9,10^ motility pattern,^11^ microbial gas production,^12^ and elaboration of short-chain fatty acids^13^ and other molecules with influence on the luminal osmotic pressure and thereby also the consistency of intestinal contents.^14,15^ Thus, it seems reasonable to assume that imbalances in this fine-tuned cross-talk is one possible causative mechanism behind at least some of the symptoms and findings contributing to the clinical picture of IBS-D.

By accepting IBS as the development of a microbial imbalance or dysbiosis in the gut, it seems logical to restore the disturbed intestinal microbiota by administration of a well-balanced intestinal microbiota, either from a healthy donor, or, as in our 2 cases, a well-defined anaerobic cultivated human intestinal microbiota originating from a healthy donor.

If dysbiosis is the underlying cause of IBS-D, it is reasonable to assume that corresponding mechanisms prevail in other forms of dysfunctional gastrointestinal conditions, such as chronic constipation. Following this hypothesis, out of 45 patients that were treated with a fecal microbiota transplant 60% were able to terminate their laxative treatment.^16^

At present, antibiotic-associated diarrhea often leading to *C difficile* infection represents serious burden on the quality of life and a threat to human health, especially in developed countries. Antibiotic-associated diarrhea can be looked upon as an acute, antibiotic-induced intestinal dysbiosis. Initially, *C difficile* infection is also a dysbiosis, allowing *C difficile* to multiply and to produce toxins. The value of treating these conditions by restoration of a normal gut microbiota, either by transplantation of fresh fecal material or by various subsets of intestinal microbiota, is now well documented.^17,18^

Taken together, we suggest the introduction of the term *dysbiotic bowel syndrome* (DBS) as a new concept describing the effects and consequences of an unbalanced intestinal microbiota with related symptoms. DBS should be diagnosed on clinical grounds as an intestinal condition in which functional disturbances of the microbiota are influencing several physiological and/or biochemical parameters in the host leading to symptoms from the gut or elsewhere in the body. In cases where DBS is assumed to be present, restorative microbiota therapy should be tried. At present, the diagnosis will be based on the medical history, the presence of symptoms, and the outcome of restoration of the gut microbiota as in our 2 cases. Hopefully, future research will provide suitable biomarkers for a rapid diagnosis as well as knowledge of how to restore the disturbed microbiota, preferably with a cultured microbiota, which is virtually risk free compared to donor feces.
